# Supplementary material for: Two species of the green algae Volvox sect. Volvox from the Japanese ancient lake, Lake Biwa
Source: PLoS One. 2024 Sep 23;19(9):e0310549. doi: 10.1371/journal.pone.0310549 (PMC11419359; doi:10.1371/journal.pone.0310549)
Supplement: S2 Table — (DOCX) [file pone.0310549.s002.docx]

**S2 Table. Comparison of *Volvox biwakoensis* sp. nov. and previously described bisexual/monoicous morphological type and species of *Volvox* sect. *Volvox.* Based on Nozaki et al. [1].**

|  | *V. biwakoensis* sp. nov. | *V. longispi-*  *niferus* | *V. capensis* | *V. globator* | *V. merrillii* | *V. amboensis* | *V. barberi* | *V. kirkiorum* | *V. ferrisii* | *Volvox* sp. Sagami |
| --- | --- | --- | --- | --- | --- | --- | --- | --- | --- | --- |
| Size of asexual spheroid (µm) | up to 800 ^a^ | up to 830 ^a^ | up to 1323 × up to 1357 | 380–500 × 400–575 | 670–890 × 710–925 | 800–1300 | 525–880 × 575–910 | 400–790 × 430–930 ^a^ | 630–1000 × 650–1000 ^a^ | up to 590 |
| Number of cells in asexual spheroid | 6000-9000 | 5600–8200 | 2000–23000 | 8000–17000 | 9800–20100 | 30000–50000 | 35000–47800 | 3000–6000 | 5000–8000 | 2000–10000  (usually 2000–5000) |
| Number of gonidia in asexual spheroid | 4-10 (usually 5-8) | 7–11 | 4–20 (usually 6–10) | 3–17 (usually 4–7) | 3–14 (usually 4–7) | 1–14 (usually 8) | 3–9 | 2–8 (usually 4–6) | 2–8 (usually 3–5) | 4–8 |
| Size of sexual colony (µm) | up to 350 | up to 800 ^a^ | up to 1292 × up to 1335 | 350–480 × 370–510 | 375–650 × 390–690 | up to 2000 | 550–880 × 605–920 | 430–440 × 510–540 ^a^ | 600–800 × 650–900 ^a^ | up to 500 |
| Number of cells in sexual spheroid | 3600-5000 | 4900–7800 | 3200–6200 or more | 9400–17500 | 12500–23000 |  | 23200–53100 | 1000–6000 | 5000–13000 | 4000–8000 |
| Number of zygotes (eggs) in sexual spheroid | 4-26 | 25–58 | 39–160  [50–220 ^b^]  [usually 70–100 ^c^] | 11–70 (usually 20–30) | 45–174 (usually 60–130) | 20–700 (usually more than 200) | 72–251 (usually 100–200) | 20–100 (usually 30–50) | 70–250 (usually 100–150) | 5–25 (usually 10–20) |
| Number of sperm packets in sexual spheroid | 4-8 | 5–16 | 2–19  [7–35 ^b^]  [2–6 ^c^] | 3–7 | 5–8 |  | 4–8 | 4–8 | 3–5 | 1–5 |
| Shape of anterior somatic cells | ovoid to ellipsoidal with a narrow anterior face | sub- spherical or trapezoidal | pear-  shaped or ovoid | flattened | flattened (wider than high) | pear-  shaped | elongate- ovoid or elongate- ellipsoidal | pear- shaped to ovoid | ellipsoidal to ovoid | elongate- ovoid or elongate- ellipsoidal |
| Diameter of zygotes without spines (µm) | 46-50 | 40–48 | 32–49  [40–53 ^b^] | 36–55 | 37–40 | 30–37 | 34–40 | 32–38 | 35–40 | 37–48 |
| Shape of spines of zygote | straight or slightly curved with acute apices | straight or slightly curved with acute apices | straight with rounded or blunt apices | straight with rounded apices | curved with acute apices | curved with acute apices | straight with acute apices | straight with acute apices | straight with acute apices | strait with acute apices |
| Length of spines of zygotes (µm) | 5-7 | 12–14 | 4–8  [7–13 ^b^] | 3–8 | ca. 11 | 5–7 | 3–5.5 | 5.5–8 | 6–8.5 | up to 3 |
| Distribution | Lake Biwa, Japan | Thailand | South Africa, USA | Europe, USA, India | Philippine, India, Australia | Namibia, Africa | Philippine, USA, New Zealand | Japan | Japan | Japan |
| References | the present study | Nozaki et al. [1] | Rich and Pocock [2], Smith [3], Starr et al. [4], Nozaki et al. [5] | Shaw [6], Rich and Pocock [2], Smith [3], Pocock [7], Isaka et al. [8] | Shaw [6], Smith [3], Cave and Pocock [9] | Rich and Pocock [2], Smith [3] | Shaw [6],  Smith [3],  Pocock [7], Isaka et al. [8] | Isaka et al. [8], the present study | Isaka et al*.* [8] | Nozaki et al. [10], the present study |

^a^ Sizes of fully matured spheroids

^b^ *V. capensi*s f. *rhodesiensis* [2]

^c^ *V. capensis* from USA [5]

**References**

1. Nozaki H, Mahakham W, Heman W, Matsuzaki R, Kawachi M. A new preferentially outcrossing monoicous species of *Volvox* sect. *Volvox* (Chlorophyta) from Thailand. PLoS ONE 2020, 15: e0235622. doi: 10.1371/journal.pone.0235622

2. Rich F, Pocock MA. Observations on the genus *Volvox* in Africa. Ann. S. Afr. Mus. 1933; 16: 427–471, pls. 9–24.

3. Smith GM. A comparative study of the species of *Volvox*. Trans. Am. Microsc. Soc. 1944; 63: 265–310.

4. Starr RC, O'neil RM, Miller CE. L-Glutamic acid as a mediator of sexual morphogenesis in *Volvox capensis*. Proc. Natl. Acad. Sci. USA 1980; 77: 1025–1028.

5. Nozaki H, Ueki N, Misumi O, Yamamoto K, Yamashita S, Herron MD, et al. Morphology and reproduction of *Volvox capensis* (Volvocales, Chlorophyceae) from Montana, USA. Phycologia 2015; 54: 316–320. doi: 10.2216/15-14.1

6. Shaw WR. *Janetosphaera*, a new genus, and two new species of *Volvox*. Philipp. J. Sci. 1922; 20: 477–508, plates 1–5.

7. Pocock MA. Notes on the occurrence in New Zealand of *Volvulina steinii* Playfair and species of *Volvox.* Linn. Rec. Cant. Mus. 1951; 6: 1-8. https://eurekamag.com/research/023/187/023187130.php

8. Isaka N, Kawai-Toyooka H, Matsuzaki R, Nakada T, Nozaki H. Description of two new monoecious species of *Volvox* sect. *Volvox* (Volvocaceae, Chlorophyceae), based on comparative morphology and molecular phylogeny of cultured material. J. Phycol. 2012; 48: 759–767. doi: 10.1111/j.1529-8817.2012.01142.x

9. Cave MS, Pocock MA. Karyological studies in the Volvocaceae. Am. J. Bot. 1951; 38: 800-811. doi: 10.2307/2438205

10. Nozaki H, Ueki N, Isaka N, Saigo T, Yamamoto K, Matsuzaki R, et al. A New Morphological Type of *Volvox* from Japanese Large Lakes and Recent Divergence of this Type and *V*. *ferrisii* in Two Different Freshwater Habitats. PLoS ONE 2016, 11: e0167148. doi.org/10.1371/journal.pone.0167148
