# Supplementary material for: Two species of the green algae Volvox sect. Volvox from the Japanese ancient lake, Lake Biwa
Source: PLoS One. 2024 Sep 23;19(9):e0310549. doi: 10.1371/journal.pone.0310549 (PMC11419359; doi:10.1371/journal.pone.0310549)
Supplement: S1 Fig — (DOCX) [file pone.0310549.s003.docx]

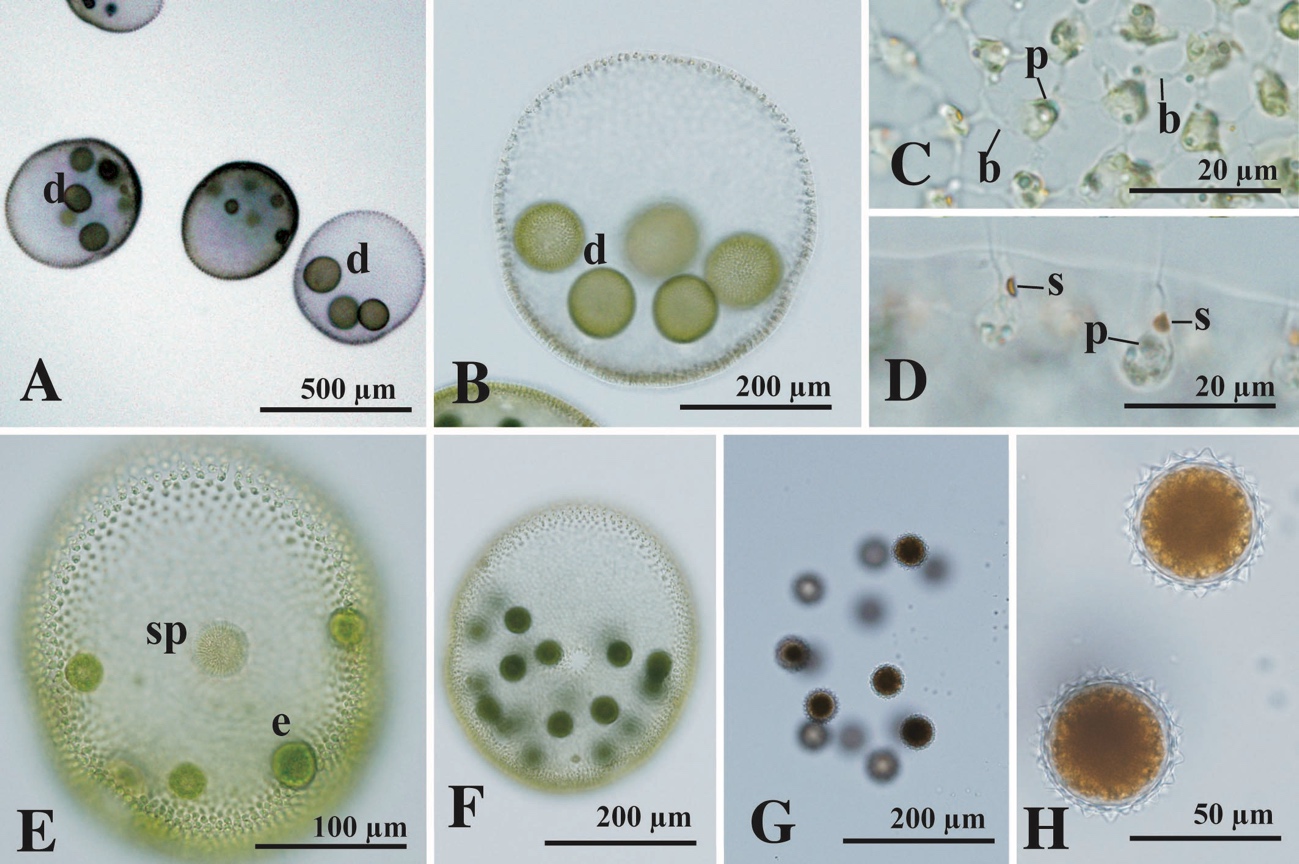


**S1 Fig. Bright-field microscopy of *Volvox* sp. Sagami strain SB01 from Tokyo, Japan.**

(A, B) Asexual spheroids with developing embryos or daughter spheroids (d). (C, D) Part of asexual spheroids showing somatic cells. (C) Transverse section of somatic cells showing cytoplasmic bridges (b) and pyrenoid (p) in the chloroplast. (D) Side view of anterior somatic cells showing stigma (s) and pyrenoid (p) in the chloroplast. (E–H) Sexual reproduction. (E) Sexual spheroid with eggs (e) and sperm packets (sp). (F) Sexual spheroid with immature zygotes. (G) Sexual spheroid with matured zygotes. (H) Matured zygotes with short spines on zygote walls.
