# Supplementary material for: Two species of the green algae Volvox sect. Volvox from the Japanese ancient lake, Lake Biwa
Source: PLoS One. 2024 Sep 23;19(9):e0310549. doi: 10.1371/journal.pone.0310549 (PMC11419359; doi:10.1371/journal.pone.0310549)
Supplement: S2 Fig — (DOCX) [file pone.0310549.s004.docx]

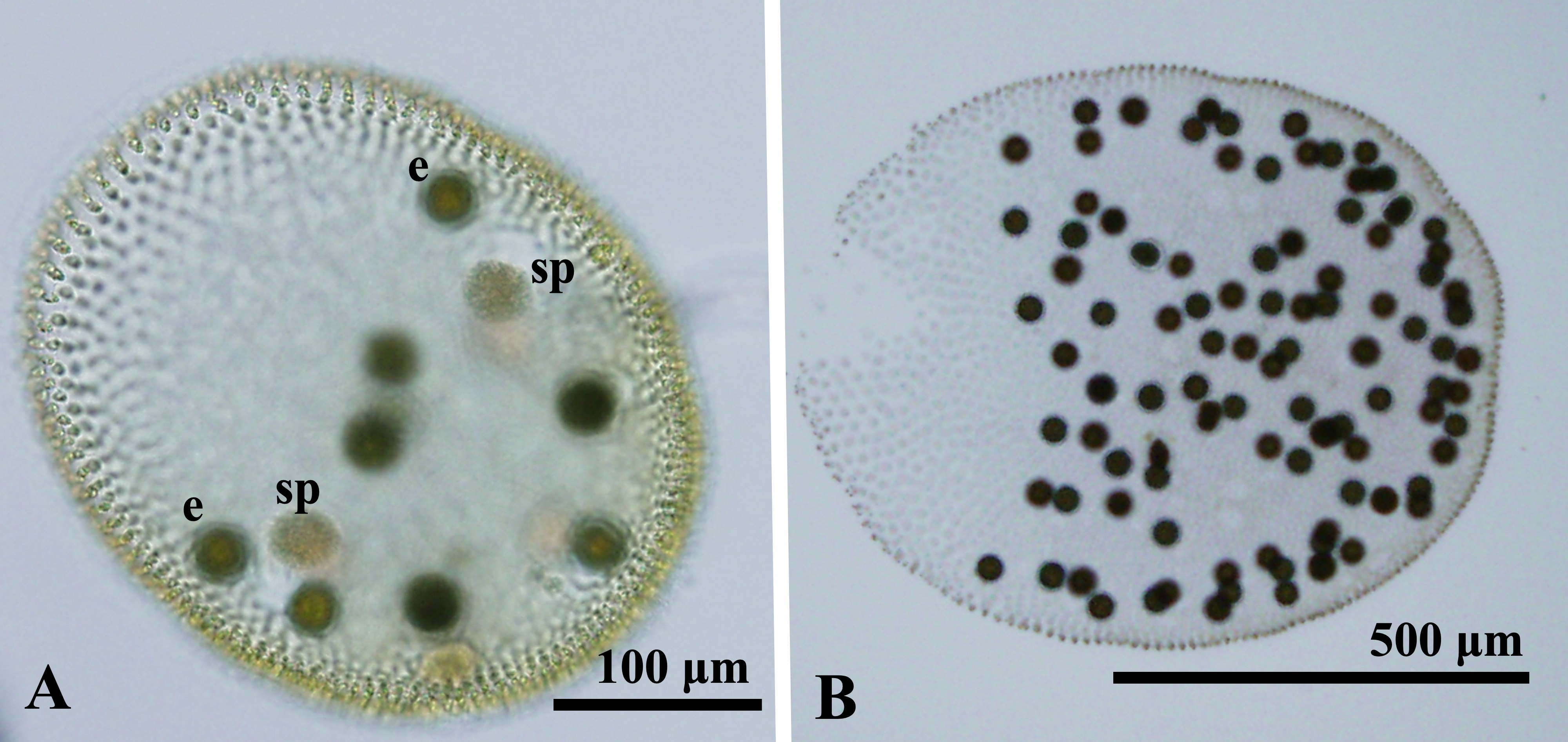


**S2 Fig. Bright-field microscopy of *Volvox kirkiorum* from Lake Biwa, Japan.**

(A) Small bisexual or monoicous sexual spheroid with 10 eggs and six sperm packets. Strain 2022-1027-VVx9. (B) Squashed mature sexual spheroid showing approximately 100 zygotes. Strain 2022-VVx11.
