# Supplementary material for: Two species of the green algae Volvox sect. Volvox from the Japanese ancient lake, Lake Biwa
Source: PLoS One. 2024 Sep 23;19(9):e0310549. doi: 10.1371/journal.pone.0310549 (PMC11419359; doi:10.1371/journal.pone.0310549)
Supplement: S3 Fig — (DOCX) [file pone.0310549.s005.docx]

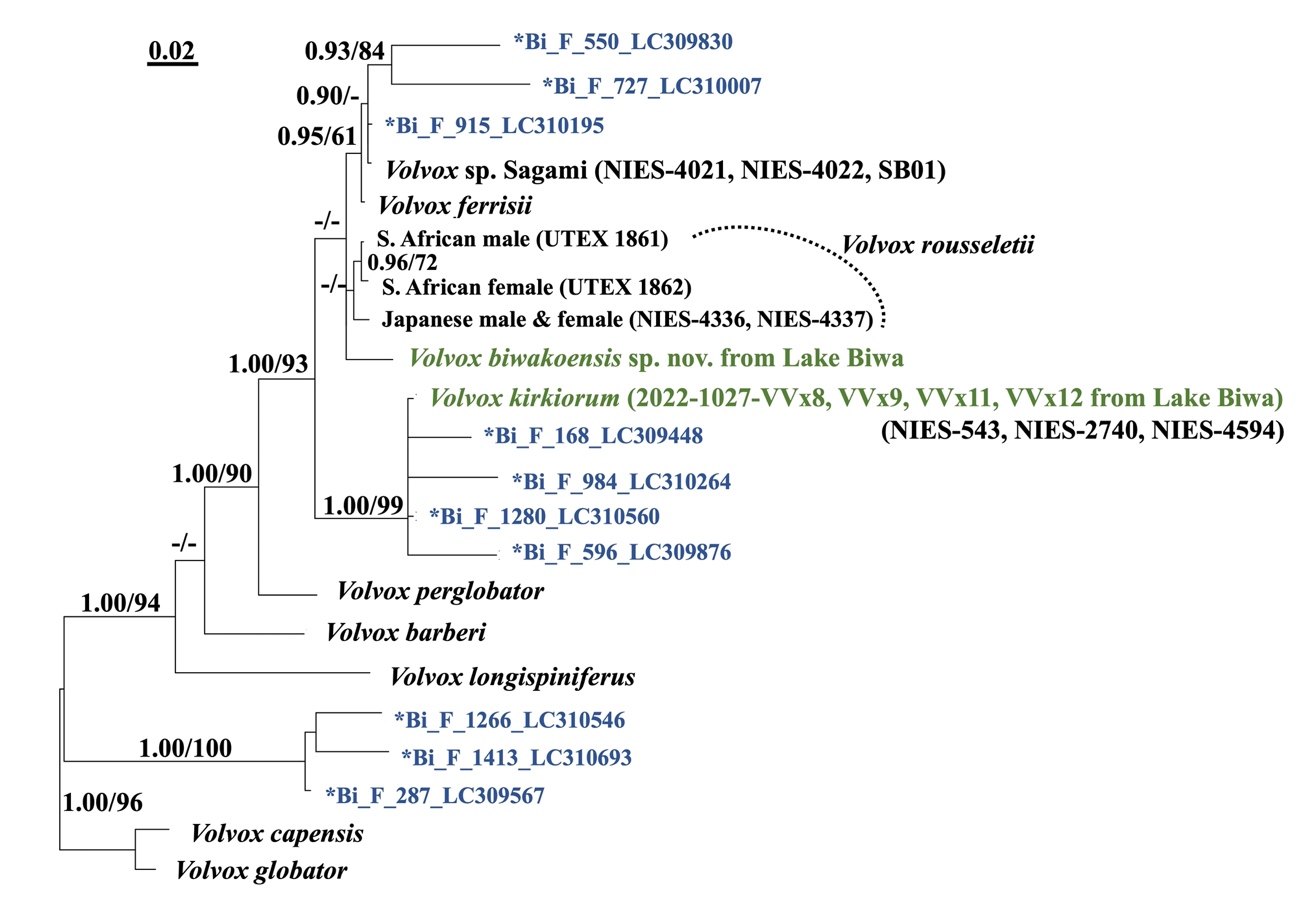


**S3 Fig. Phylogeny of *Volvox* sect. *Volvox* and its related metabarcoding sequences (blue) based on the Internal Transcribed Spacer (ITS) regions of nuclear ribosomal DNA (rDNA) (ITS-1, 5.8S rDNA, and ITS-2).**

For alignment data, see S3 Appendix. Ten metabarcoding sequences targeting ITS2 rDNA regions from Lake Biwa samples [1] were added to the data matrix of Fig. 1. The tree was constructed based on Bayesian Inference (BI) with K80+G model. Branch lengths represent the evolutionary distances shown by the scale bar. Posterior probabilities (0.90 or more) by BI and bootstrap values (50% or more) based on 1000 replications of maximum likelihood method (with K80+G model) are described by numbers in left and right sides at branches, respectively.

**Reference**

1. Song P, Tanabe S, Yi R, Kagami M, Liu X, Ban S. Fungal community structure at pelagic and littoral sites in Lake Biwa determined with high-throughput sequencing. Limnology. 2018: 19: 241–251. doi: 10.1007/s10201-017-0537-8
