# Supplementary material for: Two species of the green algae Volvox sect. Volvox from the Japanese ancient lake, Lake Biwa
Source: PLoS One. 2024 Sep 23;19(9):e0310549. doi: 10.1371/journal.pone.0310549 (PMC11419359; doi:10.1371/journal.pone.0310549)
Supplement: Attachment — Submitted filename: Review_Manuscript Number PONE-D-24-23039.docx [file pone.0310549.s009.docx]

I support the publication of this article after minor revision. In terms of its content and illustrations (figures and tables), the article is at a very high scientific level. I only have two critical comments.

1. It is advisable to provide additional information about the ancient Lake Biwa: the surface area of the lake, its average and maximum depth and the elevation of the surface of the lake above sea level.

2. The second critical comment concerns supporting information: in S2 Table (Comparison of *Volvox biwakoensis* sp. nov. and previously described bisexual/monoicous morphological type and species of *Volvox* sect. *Volvox*) the data presented on the geographical distribution of several species of *Volvox* are incomplete. It is necessary to add information obtained by Mary Pocock (the recognized expert in this field) about *Volvox* in Australia and New Zealand:

*Volvox merrillii* is known not only from the Philippines and India (as listed in the S2 Table), but also from Australia (New South Wales) - M.S. Cave & M.A. Pocock. Karyological Studies in the Volvocaceae. American Journal of Botany, Vol. 38, No. 10 (Dec., 1951), pp. 800-811.

Regarding the distribution of *Volvox barberi* and *Volvox globator*, it should be added that Pocock found these two species in New Zealand - Pocock M.A. Notes on the occurrence in New Zealand of *Volvulina steinii* Pleyfair and species of *Volvox* Linn. Rec. Cant. Mus., 6: 1. This hard-to-find article is cited in: Chapman VJ, Thompson RH, Segar ECM (1957) Check list of fresh-water algae of New Zealand. Trans. Roy. Soc. New Zealand. 84(4): 695–747.
