# Supplementary material for: Two species of the green algae Volvox sect. Volvox from the Japanese ancient lake, Lake Biwa
Source: PLoS One. 2024 Sep 23;19(9):e0310549. doi: 10.1371/journal.pone.0310549 (PMC11419359; doi:10.1371/journal.pone.0310549)
Supplement: Attachment — Submitted filename: Response to Reviewers -ok2.docx [file pone.0310549.s010.docx]

Dear Dr. Michael Klymkowsky,

Thank you very much for your scholarly and positive decision of our manuscript entitled " **Two species of the green algae *Volvox* sect. *Volvox* from the Japanese ancient lake, Lake Biwa**" for publication in ***PLOS ONE*** as a research article.

Based on the comments/suggestions raised by the two reviewers, we have revised the manuscript completely. Our responses to the comments have been described in the followings in red pen.

Sincerely,

Hisayoshi Nozaki

PONE-D-24-23039

Two species of the green algae Volvox sect. Volvox from the Japanese ancient lake, Lake Biwa

PLOS ONE

Dear Dr. Nozaki,

Thank you for submitting your manuscript to PLOS ONE. After careful consideration, we feel that it has merit but does not fully meet PLOS ONE’s publication criteria as it currently stands. Therefore, we invite you to submit a revised version of the manuscript that addresses the points raised during the review process.

Please submit your revised manuscript by Aug 22 2024 11:59PM. If you will need more time than this to complete your revisions, please reply to this message or contact the journal office at <a href="mailto:plosone@plos.org">plosone@plos.org. When you're ready to submit your revision, log on to https://www.editorialmanager.com/pone/ and select the 'Submissions Needing Revision' folder to locate your manuscript file.

Please include the following items when submitting your revised manuscript:

- A rebuttal letter that responds to each point raised by the academic editor and reviewer(s). You should upload this letter as a separate file labeled 'Response to Reviewers'.
- A marked-up copy of your manuscript that highlights changes made to the original version. You should upload this as a separate file labeled 'Revised Manuscript with Track Changes'.
- An unmarked version of your revised paper without tracked changes. You should upload this as a separate file labeled 'Manuscript'.

Response: These three files have been uploaded. In addition, S2 Table with tracked changes and S3 Figure with tracked changes have also prepared, in addition to the unmarked revised version.

If you would like to make changes to your financial disclosure, please include your updated statement in your cover letter. Guidelines for resubmitting your figure files are available below the reviewer comments at the end of this letter.

Response: As suggested, all funding-related text has been removed from ‘Financial Disclosure’ sections of the revised manuscript. Figures 1-5 have been revised by using PACE and five tiff files of Figures 1-5 have been uploaded.

If applicable, we recommend that you deposit your laboratory protocols in protocols.io to enhance the reproducibility of your results. Protocols.io assigns your protocol its own identifier (DOI) so that it can be cited independently in the future. For instructions see: <https://journals.plos.org/plosone/s/submission-guidelines#loc-laboratory-protocols>. Additionally, PLOS ONE offers an option for publishing peer-reviewed Lab Protocol articles, which describe protocols hosted on protocols.io. Read more information on sharing protocols at <https://plos.org/protocols?utm_medium=editorial-email&utm_source=authorletters&utm_campaign=protocols>.

We look forward to receiving your revised manuscript.

Kind regards,

Michael Klymkowsky, Ph.D.
Academic Editor
PLOS ONE

Journal Requirements:

When submitting your revision, we need you to address these additional requirements.

1. Please ensure that your manuscript meets PLOS ONE's style requirements, including those for file naming. The PLOS ONE style templates can be found at 
https://journals.plos.org/plosone/s/file?id=wjVg/PLOSOne_formatting_sample_main_body.pdf and 
https://journals.plos.org/plosone/s/file?id=ba62/PLOSOne_formatting_sample_title_authors_affiliations.pdf

Response: I have ensured that our manuscript meets PLOS ONE's style requirements, including those for file naming.

2. "We noticed you have some minor occurrence of overlapping text with the following previous publication(s), which needs to be addressed:

https://doi.org/10.1371/journal.pone.0235622

In your revision ensure you cite all your sources (including your own works), and quote or rephrase any duplicated text outside the methods section. Further consideration is dependent on these concerns being addressed."

Response: In the description of the holotype, the sentences have been changed. In addition, figure legends of Figures 1, 2, and S3 Figure have been revised in explanation of BI and bootstrap values and branch lengths. However, "Nomenclature" has not been changed because it belongs to the methods section. In S2 Table, Nozaki et al. [1] (https://doi.org/10.1371/journal.pone.0235622) was already cited in the top of the table.

3. Please note that funding information should not appear in any section or other areas of your manuscript. We will only publish funding information present in the Funding Statement section of the online submission form. Please remove any funding-related text from the manuscript.

Response: All funding-related text has been removed from the revised manuscript.

4. We note that the grant information you provided in the ‘Funding Information’ and ‘Financial Disclosure’ sections do not match.

When you resubmit, please ensure that you provide the correct grant numbers for the awards you received for your study in the ‘Funding Information’ section.

Response: As described above, all funding-related text has been removed from the main manuscript of revised version.

The correct grant numbers for the awards we received for our study have been described in the ‘Funding Information’

as follows:

JSPS KAKENHI

Award Number: 19K22446, 24K08946 | Recipient: Hisayoshi Nozaki, Ph.D.

JSPS KAKENHI

Award Number: 21K06295 | Recipient: Noriko Ueki, Ph.D.

The Collaborative Research Fund from Shiga Prefecture entitled “Study on water quality and lake-bottom environment for protection of the soundness of water environment” under the Japanese Grant for Regional Revitalization

Award Number: OS2024RR1 | Recipient: Koichi Shimotori, Ph.D.

Network Joint Research Center for Materials and Devices

Award Number: 20211204, 20221238, 20231201, 20241233 | Recipient: Noriko Ueki, Ph.D.

We prepared Financial Disclosure as suggested*. However, because of the possible error of the editorial manager system of PLOS ONE, we cannot access 'Financial Disclosure' of Additional Information via the editorial manager system.

Please help me!

*This study was partially supported by a Grants-in-Aid for Scientific Research (grant numbers 19K22446 and 24K08946 for HN, 21K06295 to NU) from the Ministry of Education, Culture, Sports, Science and Technology (MEXT)/Japan Society for the Promotion of Science (JSPS) KAKENHI (<https://www.jsps.go.jp/english/e-grants/>), the Collaborative Research Fund (grant number OS2024RR1 to KS) from Shiga Prefecture entitled “Study on water quality and lake-bottom environment for protection of the soundness of water environment” under the Japanese Grant for Regional Revitalization (https://www.mlit.go.jp/en/index.html), and the Research Program of "Dynamic Alliance for Open Innovation Bridging Human, Environment and Materials" (grand numbers 20211204, 20221238, 20231201 and 20241233 for NU) in "Network Joint Research Center for Materials and Devices" (https://alliance.tagen.tohoku.ac.jp/english/). The funders had no role in study design, data collection and analysis, decision to publish, or preparation of the manuscript.

5. When completing the data availability statement of the submission form, you indicated that you will make your data available on acceptance. We strongly recommend all authors decide on a data sharing plan before acceptance, as the process can be lengthy and hold up publication timelines. Please note that, though access restrictions are acceptable now, your entire data will need to be made freely accessible if your manuscript is accepted for publication. This policy applies to all data except where public deposition would breach compliance with the protocol approved by your research ethics board. If you are unable to adhere to our open data policy, please kindly revise your statement to explain your reasoning and we will seek the editor's input on an exemption. Please be assured that, once you have provided your new statement, the assessment of your exemption will not hold up the peer review process.

Response: I have asked DDBJ to release the new sequence data immediately. The DDBJ/ENA/GenBank accession numbers (LCLC817957–LC817993 and LC818981) are now available.

6.  Please review your reference list to ensure that it is complete and correct. If you have cited papers that have been retracted, please include the rationale for doing so in the manuscript text, or remove these references and replace them with relevant current references. Any changes to the reference list should be mentioned in the rebuttal letter that accompanies your revised manuscript. If you need to cite a retracted article, indicate the article’s retracted status in the References list and also include a citation and full reference for the retraction notice.

Response: A book [13] has been added to reference the details of Lake Biwa (see comment 1 of Reviewer #2) and the divergence has been discussed based on the major comment by reviewer#1 by citing [26]. :

13. Kawanabe H, Nishino M, Maehata M, editors. Lake Biwa: interactions between nature and people. New York: Springer; 2012. doi: 10.1007/978-94-007-1783-1

26. Yamagishi S, Yamamoto K, Takahashi K, Kawai-Toyooka H, Suzuki S, Matsuzaki R, Yamaguchi H, Kawachi M, Higashiyama T, Nozaki H. Evolutionary analysis of *MID* homologs during the transition from homothallic species to heterothallic species in *Volvox* sect. *Volvox* (Chlorophyceae). Phycol. Res. 2024; 72: 46-55. [doi: 10.1111/pre.12538](https://doi.org/10.1111/pre.12538)

Additional Editor Comments:
**Both reviewers call for minor revisions that expect with be simple to address.** 

[Note: HTML markup is below. Please do not edit.]

Reviewers' comments:

Reviewer's Responses to Questions

**Comments to the Author**

1. Is the manuscript technically sound, and do the data support the conclusions?

The manuscript must describe a technically sound piece of scientific research with data that supports the conclusions. Experiments must have been conducted rigorously, with appropriate controls, replication, and sample sizes. The conclusions must be drawn appropriately based on the data presented.

Reviewer #1: Yes

Reviewer #2: Yes

2. Has the statistical analysis been performed appropriately and rigorously?

Reviewer #1: Yes

Reviewer #2: N/A

3. Have the authors made all data underlying the findings in their manuscript fully available?

The [PLOS Data policy](http://www.plosone.org/static/policies.action#sharing) requires authors to make all data underlying the findings described in their manuscript fully available without restriction, with rare exception (please refer to the Data Availability Statement in the manuscript PDF file). The data should be provided as part of the manuscript or its supporting information, or deposited to a public repository. For example, in addition to summary statistics, the data points behind means, medians and variance measures should be available. If there are restrictions on publicly sharing data—e.g. participant privacy or use of data from a third party—those must be specified.

Reviewer #1: Yes

Reviewer #2: Yes

4. Is the manuscript presented in an intelligible fashion and written in standard English?

PLOS ONE does not copyedit accepted manuscripts, so the language in submitted articles must be clear, correct, and unambiguous. Any typographical or grammatical errors should be corrected at revision, so please note any specific errors here.

Reviewer #1: Yes

Reviewer #2: Yes

5. Review Comments to the Author

Please use the space provided to explain your answers to the questions above. You may also include additional comments for the author, including concerns about dual publication, research ethics, or publication ethics. (Please upload your review as an attachment if it exceeds 20,000 characters)

Reviewer #1: The manuscript "Two species of the green algae Volvox sect. Volvox from the Japanese ancient lake, Lake Biwa" by Nozaki et al. identifies two strains of Volvox sect. Volvox including a new species from Lake Biwa as an ancient lake, which is potentially an important source of ecology and biodiversity studies but lacks the taxonomic identification of Volvox sec. Volvox at the species level worldwide. Therefore the new species Volvox biwakoensis sp. nov. with distinct zygote morphology and number among the related species should shed light on the natural history of aquatic microorganisms in ancient lakes. The authors clearly and fully described the strains they established for the publication.

Major comment

In the introduction, the manuscript discusses species diversity in ancient lakes and presents the discovery of a new species in one such lake. However, the subsequent sections do not adequately address how this discovery contributes to our understanding of species diversity in ancient lakes. Does the "possible endemism" of Volvox biwakoensis (p.15 l.320) reflect the geological history of Lake Biwa?

Response: Yes, the "possible endemism" of *Volvox biwakoensis* (p.15 l.320) may reflect the geological history of Lake Biwa. Thus, several sentences of discussion have been added to the conclusion section in the revised manuscript.

Minor comments

p.4 ll.95-6
"the south basin (Nanko) of Lake Biwa" is appropriate because Nanko is not a separate lake from Lake Biwa.

Response: Revised as suggested. Thank you!

p.12 l.257
Was germination attempted without success or it was not attempted?

Response: It was not attempted because of lack of abundant matured zygotes for induction of zygote germination. The description of the text has been revised: "Induction of zygote germination was not attempted."

p.15 l.331
"thick" instead of "chick"

Response: Revised as suggested. Thank you!

Reviewer #2: I support the publication of this article after minor revision. In terms of its content and illustrations (figures and tables), the article is at a very high scientific level. I only have two critical comments.
1. It is advisable to provide additional information about the ancient Lake Biwa: the surface area of the lake, its average and maximum depth and the elevation of the surface of the lake above sea level.

Response: These data have been added to the Method section of the revised manuscript.

2. The second critical comment concerns supporting information: in S2 Table (Comparison of Volvox biwakoensis sp. nov. and previously described bisexual/monoicous morphological type and species of Volvox sect. Volvox) the data presented on the geographical distribution of several species of Volvox are incomplete. It is necessary to add information obtained by Mary Pocock (the recognized expert in this field) about Volvox in Australia and New Zealand:
Volvox merrillii is known not only from the Philippines and India (as listed in the S2 Table), but also from Australia (New South Wales) - M.S. Cave & M.A. Pocock. Karyological Studies in the Volvocaceae. American Journal of Botany, Vol. 38, No. 10 (Dec., 1951), pp. 800-811.
Regarding the distribution of Volvox barberi and Volvox globator, it should be added that Pocock found these two species in New Zealand - Pocock M.A. Notes on the occurrence in New Zealand of Volvulina steinii Pleyfair and species of Volvox Linn. Rec. Cant. Mus., 6: 1. This hard-to-find article is cited in: Chapman VJ, Thompson RH, Segar ECM (1957) Check list of fresh-water algae of New Zealand. Trans. Roy. Soc. New Zealand. 84(4): 695–747.

Responses: Thank you very much for your very valuable and helpful information of distribution of *Volvox* sect. *Volvox*. These data have been described in S2Table by citing the two references, Pocock (1951), Cave & Pocock (1951).

6. PLOS authors have the option to publish the peer review history of their article ([what does this mean?](https://journals.plos.org/plosone/s/editorial-and-peer-review-process#loc-peer-review-history)). If published, this will include your full peer review and any attached files.

If you choose “no”, your identity will remain anonymous but your review may still be made public.

**Do you want your identity to be public for this peer review?** For information about this choice, including consent withdrawal, please see our [Privacy Policy](https://www.plos.org/privacy-policy).

Reviewer #1: No

Reviewer #2: No

[NOTE: If reviewer comments were submitted as an attachment file, they will be attached to this email and accessible via the submission site. Please log into your account, locate the manuscript record, and check for the action link "View Attachments". If this link does not appear, there are no attachment files.]

While revising your submission, please upload your figure files to the Preflight Analysis and Conversion Engine (PACE) digital diagnostic tool, <https://pacev2.apexcovantage.com/>. PACE helps ensure that figures meet PLOS requirements. To use PACE, you must first register as a user. Registration is free. Then, login and navigate to the UPLOAD tab, where you will find detailed instructions on how to use the tool. If you encounter any issues or have any questions when using PACE, please email PLOS at <a href="mailto:figures@plos.org">figures@plos.org. Please note that Supporting Information files do not need this step.
